# Pathophysiology and Immune Dysfunction in Endometriosis

**DOI:** 10.1155/2015/795976

**Published:** 2015-07-12

**Authors:** Soo Hyun Ahn, Stephany P. Monsanto, Caragh Miller, Sukhbir S. Singh, Richard Thomas, Chandrakant Tayade

**Affiliations:** ^1^Department of Biomedical and Molecular Sciences, Queen's University, Kingston, ON, Canada K7L 3N6; ^2^Department of Obstetrics and Gynecology, University of Ottawa, The Ottawa Hospital, ON, Canada K1H 7W9; ^3^Department of Obstetrics and Gynecology, Kingston General Hospital, Kingston, ON, Canada K7L 3N6

## Abstract

Endometriosis is an estrogen-dependent, chronic, proinflammatory disease prevalent in 10% of women of reproductive age worldwide. Characterized by the growth of endometrium-like tissue in aberrant locations outside of the uterus, it is responsible for symptoms including chronic pelvic pain, dysmenorrhea, and subfertility that degrade quality of life of women significantly. In Canada, direct and indirect economic cost of endometriosis amounts to 1.8 billion dollars, and this is elevated to 20 billion dollars in the United States. Despite decades of research, the etiology and pathophysiology of endometriosis still remain to be elucidated. This review aims to bring together the current understanding regarding the pathogenesis of endometriosis with specific focus on mechanisms behind vascularization of the lesions and the contribution of immune factors in facilitating lesion establishment and development. The role of hormones, immune cells, and cytokine signaling is highlighted, in addition to discussing the current pharmaceutical options available for management of pain symptoms in women with endometriosis.

## 1. D:\Finalization\CRIC\640795\texIntroduction

Endometriosis is a gynaecological condition characterized by the growth of endometrium-like tissues within and outside of the pelvic cavity. Almost 50% of adolescents with intractable dysmenorrhea or pelvic pain and 4% of women undergoing tubal ligation are diagnosed with endometriosis [1]. It has been well established that many women have a delay in diagnosis of endometriosis despite having significant dysmenorrhea and the other related symptomatology starting at a young age [2]. An important factor that contributes to the diagnostic delay is the lack of noninvasive methods for detecting endometriosis. Although endometriosis can be asymptomatic, chronic pelvic pains that are aggravated during the period of menstruation, as well as subfertility, prompt women to seek help. Based on scientific evidence that endometriosis is dependent on estrogen for growth, current pharmaceutical interventions focus on estrogen inhibition by means of either contraceptive usage or the use of drugs that inhibit ovarian secretion of estrogen. These interventions have been effective in managing pain and diminishing endometriotic lesions to some extent. However, the high rate of recurrence of endometriosis after pharmaceutical treatment or surgical ablation of the lesions drives researchers to seek other therapeutics that can effectively treat endometriosis, in terms of both symptom resolution and cure from the disease.

In this review we consolidate the current knowledge regarding the pathogenesis of endometriosis with specific focus on the mechanisms behind lesion vascularization and the contribution of immune factors in facilitating lesion development. We also focus on progesterone resistance and the role of estradiol in endometriosis. Lastly, key successful pharmaceutical interventions in improvement of symptoms commonly associated with endometriosis are discussed.

## 2. Current Theories on Endometrial Lesion Establishment

The most widely accepted theory on the pathogenesis of endometriosis is Sampson's theory of retrograde menstruation. This theory proposes that viable endometrial tissue is disseminated into the peritoneal cavity via the fallopian tubes during menstruation and subsequently implants onto peritoneal tissue or pelvic organs [3, 4]. Although only 1–10% of women are diagnosed with endometriosis, it has been found that 76–90% of healthy women undergo retrograde menstruation, as seen during laparoscopy at the menstrual or perimenstrual period [5, 6]. While increased menstrual efflux in women with endometriosis may predispose them into developing endometriosis, it is likely that women with disease suffer from fundamental differences in genetic, immunological, or biochemical factors that contribute to the development of endometriosis. Evidence for Sampson's theory comes from women with cervical stenosis and other congenital outflow obstructions. These women have an increased risk of developing endometriosis [7, 8]. This observation was recapitulated in a baboon model of endometriosis with experimentally induced cervical stenosis [9], possibly from increase in retrograde menstruation. Additionally, intraperitoneal injection of menstrual endometrium has been shown to successfully induce peritoneal endometriosis in the baboon model, with 3 out of 4 of the baboons in the study showing laparoscopically confirmed lesion progression after 12 months [10]. Despite multiple lines of evidence favoring this theory, cases of endometriosis in premenarchal girls, newborns, and males all demand secondary explanations [11].

The coelomic metaplasia theory postulates that endometriosis arises from the metaplasia of cells lining the visceral and abdominal peritoneum following various hormonal, environmental, or infectious stimuli. The basis for this theory lies in embryological studies revealing that the abdominal, pelvic, and thoracic peritoneum, the Mullerian ducts, and the germinal epithelium of the ovary are all derived from the coelomic wall epithelium. Since the cellular material that comprises the peritoneum and endometrium shares common embryonic origin—that is, the coelomic epithelium—there is a chance that the aforementioned stimuli may trigger the transformation of peritoneum into endothelial cell types. This theory may provide explanations to the above-mentioned cases of endometriosis that are inadequately explained by the theory of retrograde menstruation as well as cases of endometriosis in ectopic sites such as the lungs. Despite this, metastasis is a phenomenon that increases with age and as such does not adequately explain the drastic decline in the incidence of endometriosis following menopause in older women [11, 12]. Similarly, the embryonic rest theory proposes that the lesions arise from cells remaining from Mullerian duct migration during embryonic development following a specific stimulus such as estrogen, which plays a crucial role in the pathogenesis of endometriosis [13].

More recently, the stem cell theory has garnered much attention as several lines of experimental evidence showed the participation of both endometrial stem/progenitor cells and bone marrow-derived stem cells in the pathogenesis of endometriosis. It is believed that endometrial stem/progenitor cells from the basalis layer of the endometrium can travel via retrograde menstruation, lymphatic or vascular dissemination into the peritoneal cavity to develop into endometriotic lesions. The enhanced proliferative capacity of the stem cell and their ability to differentiate into multiple cell types may then give these cells a selective advantage in the establishment and progression of the lesion [12]. Leyendecker et al. [14] found that not only are the expressions of the estrogen receptor, the progesterone receptor, and aromatase P450 paralleled in the basalis layer and the ectopic endometrial lesion, but also endometrial fragments of the basalis layer are shed with a higher rate in women with endometriosis. Hematogenous dissemination of bone marrow-derived stem cells may also contribute to the pathogenesis of endometriosis. In one experiment, hysterectomized LacZ transgenic mice were experimentally induced with peritoneal endometriosis and then given bone marrow transplantation with cells from a LacZ transgenic mouse. LacZ expressing cells were then found in the ectopic lesion, demonstrating the potential participation of the bone marrow stem cells in the origin and persistence of the disease [15]. The stem cell theory offers an explanation for the exceptions that other theories cannot offer and demonstrates great potential as a theory describing the pathogenesis of endometriosis.

Following translocation of the endometrial tissue into the peritoneal cavity, the endometrial fragments must survive the defenses of the body, attach to a surface, and subsequently invade and modify the peritoneal membrane in order to establish a lesion. The eutopic endometrium of women with endometriosis has been shown to differ significantly from healthy controls. Not only are eutopic endometrial cells from women with endometriosis more resistant to cell mediated immune attack [16], but also they have been shown to have increased proliferative capacity [17] and increased aromatase expression, leading to increased estrogen concentrations, mediated by prostaglandin E_2_ [18]. These alterations may be a result of inherited or acquired genetic factors. Studies show that the risk of endometriosis is approximately six times higher when the woman has a first-degree relative with a severe form of endometriosis [19]. Polymorphisms in genes involved in detoxification processes, estrogen receptors, cytokines, immunomodulatory proteins (i.e., Toll-like receptors), and factors involved in both attachment and invasion have been studied and confirmed in women with endometriosis. Defective immune surveillance is also thought to be a contributing factor to the ability of sloughed endometrium to successfully establish into a lesion.

Attachment of endometrial tissue may be facilitated more easily with larger fragments, owing to the intact integrity of the cells and tissue composition [20]. Current knowledge suggests that endometrial stromal cells are involved in the attachment of the lesion, whereas endometrial glandular epithelial cells primarily play a role in the invasion and growth of the lesion [21]. An aberrant integrin expression profile of eutopic endometrium in women with endometriosis is thought to play a fundamental role in the implantation of the endometrial cells to the collagen types I and IV, fibronectin, vitronectin, tenascin, and laminin of the peritoneum [21].

Following attachment, degradation of the extracellular matrix (ECM) takes place, allowing endometrial cells to invade and potentially establish endometriotic foci from which the lesion will progress. The endometrium of women with endometriosis has been shown to have increased proteolytic capacity. Anomalous expressions of plasminogen activator system proteins as well as various matrix metalloproteinases (MMPs) seem to be responsible for this phenomenon [22]. Recent studies have shown that MMP-2, MMP-3, MMP-7, and MMP-9 levels are all increased in endometriosis [23]. In addition, urokinase-type plasminogen activator (uPA), which catalyzes the conversion of plasminogen to plasmin, has been shown to be elevated in the eutopic endometrium and ectopic endometriotic lesion, as well as the peritoneal fluid (PF) of women with endometriosis [22, 24]. Plasmin is involved in the degradation of ECM proteins as well as the activation of MMPs and growth factors and thus likely plays a vital role in the establishment of a lesion [24].

## 3. Increased Estradiol Production and Progesterone Resistance in Endometriosis

As discussed earlier, the most widely accepted theory of retrograde menstruation postulates the pathogenesis of endometriosis to begin with the invasion and proliferation of menstrual effluents in the PF. From there, studies suggest that aberrant immune mechanisms and responses to ovarian steroids found in only a subset of women would lead to the development of endometriotic foci in the peritoneal membrane. Interestingly, in a baboon model of endometriosis, menstrual phase endometrium injected intraperitoneally displayed enhanced adherence to the peritoneal membrane compared to the luteal phase endometrium [10]. This suggests that menstrual phase endometrial fragments express selective factors that are yet to be characterized, allowing for subsequent implantation in aberrant locations. Under normal physiological circumstances, human endometrium is under cyclical regulation by estrogen and progesterone, with the superficial, functionalis endometrial layer undergoing proliferation, differentiation, and shedding if implantation does not occur. However, the cellular components of the ectopic endometriotic lesions respond to ovarian steroids in a different manner when compared to normal eutopic endometrium [25]. Macroscopically apparent structural malformation of the endometrial epithelium of women with endometriosis lends clues to increased incidence of infertility in women with endometriosis [26] and perhaps offers an explanation as to why only a subset of women develop endometriosis.

Estradiol (E2), a biologically active form of estrogen, plays a critical role in the reconstruction of the endometrium after menstruation. Proliferation of endometrial cells and reestablishment of vasculature of the functionalis endometrial layer are driven by the actions of E2 interacting with its nuclear receptors, ER-*α* and ER-*β*. Endometrial E2 arises mainly from the ovaries and also from extraovarian tissues such as the adrenal gland and adipocytes which arrive at tissue via circulation. Aromatase P450 (aromP450) is an enzyme that catalyzes the conversion of ovarian androstenedione into estrone. From there, 17*β*-hydroxysteroid dehydrogenase type 1 (17*β*-HSDT1) further catalyzes the conversion of estrone into E2. Prostaglandin E_2_ (PGE_2_) is synthesized from arachidonic acid by the activity of rate limiting enzyme cyclooxygenase-2 (COX-2). PGE_2_ induces aromP450 production via the cAMP cell signaling cascade in the ectopic endometriotic stromal cells in a dose dependent manner [27]. In the endometrium of healthy women, the activity of aromP450 is undetectable [27]. However, both endometrium and the ectopic endometriotic lesion of women with endometriosis express this enzyme in significantly high amounts, allowing local production of E2. The ability of the lesion to produce E2* de novo*, in addition to manufacturing the enzymes required for its production, may facilitate the implantation of endometrial fragments in the peritoneal cavity [27, 28].

Due to widely implicated roles of E2 in the pathogenesis of endometriosis, a variety of pharmaceutical interventions targeting the inhibition of estrogen production are administered to women with endometriosis, but with mixed success. Most of all, the symptoms of pain may be managed while on treatment; however, pain often reappears promptly with the discontinuation of the treatment. Around half of patients using progestins reported recurrence of pelvic pain after treatment cessation [29]. Furthermore, long term usage may be deterred by the undesirable side effects consisting of breakthrough bleeding, weight gain, and bone mineral density loss from treatments including GnRH (gonadotropin releasing hormone) agonists and depot progestins (medroxyprogesterone acetate) [30]. A third-line treatment, aromatase inhibitors (AI), can be used in conjunction with other types of inhibitors targeted towards estrogen suppression. However, with some women showing development of resistance to current hormonal therapies, further investigations are needed targeting improvements to current therapeutic interventions [31].

In addition to the enhanced local production of E2 in both eutopic endometrium and ectopic endometriotic lesions in women with endometriosis, resistance to progesterone contributes to the pathogenesis of endometriosis. Progesterone, which is mainly produced during the secretory phase of the menstrual cycle, inhibits the action of estrogen and prepares the endometrium for implantation. The process of decidualization, whereby the endometrial epithelial and stromal cells begin to differentiate, is facilitated by progesterone. Similar to estrogen, progesterone interacts with two receptor isoforms, PR-A and PR-B, each with distinct functions. Gene ablation of PR-A in mice leads to uterine and ovarian abnormalities, while ablation of PR-B does not affect uterine or ovarian function [32]. Furthermore, both PR-A and PR-B transcripts are made from a single gene with a shorter PR-A transcript than PR-B, which results in the ability of PR-A to become transrepressor of PR-B and other nuclear receptors [32]. Interestingly, endometriotic lesions lack PR-B, and the transrepressor PR-A is barely expressed [33]. This is evidence that progesterone resistance in endometriosis may lie at the molecular level. Decreased responsiveness to progesterone is further substantiated by Bulun et al. [17] which showed decreased responsiveness of endometriotic stromal cells to progesterone by measuring the levels of prolactin mRNA, which is normally induced by progesterone. Treatment of endometriotic stromal cells with medroxyprogesterone acetate (MPA), a synthetic variant of progesterone, resulted in much lower levels of prolactin mRNA compared to eutopic endometrial stromal cells [17]. Such resistance to progesterone treatment ensures increased local concentration of E2 due to the inability of progesterone to activate 17*β*-hydroxysteroid dehydrogenase type 2 (17*β*-HSDT2), which catalyzes deactivation of E2 to estrone [34]. Normally, progesterone mediated factors from endometrial stromal cells induce expression of 17*β*-HSDT2 from the endometrial epithelial cells in a paracrine manner. This mechanism was suppressed in Ishikawa endometrial epithelial cell line cultured with conditioned medium from the ectopic endometriotic stromal cells [34]. Thus, studies show that, unlike eutopic endometrium, progesterone resistance is prevalent in the ectopic endometriotic lesions, which may contribute to the increased concentration of local E2 that may further promote the growth of the endometriotic lesions.

## 4. Angiogenesis and Vasculogenesis in Endometriosis

**Figure 1:**
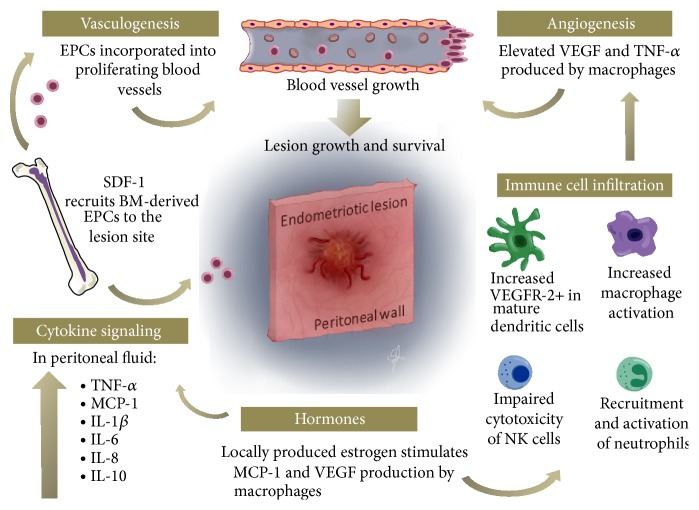
An overview of immune cells and mediators involved in the promotion of neovascularization and endometriotic lesion growth on the peritoneal membrane. In women with endometriosis, high levels of angiogenic factors and inflammatory cytokines are found in the peritoneal fluid (PF). Development of blood vessels of the lesions depends on two processes: vasculogenesis and angiogenesis. Vasculogenesis is mediated by recruitment and incorporation of the bone marrow- (BM-) derived endothelial progenitor cells (EPCs) to proliferating blood vessels in the endometriotic lesions. Recruitment of BM-derived EPCs is facilitated by stromal cell-derived factor- (SDF-) 1. Vascular endothelial growth factor (VEGF) and other angiogenic factors including interleukin- (IL-) 6, IL-8, and tumor necrosis factor- (TNF-) *α* mediate the process of angiogenesis by activating angiogenic switch of endothelial cells. Local production of estradiol by the lesion maintains the expression of VEGF and promotes the production of VEGF and monocyte chemoattractant protein- (MCP-) 1 by the macrophages. In women with endometriosis, natural killer (NK) cell cytotoxicity is diminished, which may be due to increased expression of IL-10 in the PF. Immature dendritic cells (DCs) express VEGFR-2 on the surface and thus are theorized to play a role in angiogenesis by interacting with VEGF. The integrated role of immune cells and mediators is a complicated process and requires further studies to piece together the details available to fully appreciate their role in the pathogenesis of endometriosis.

Angiogenesis refers to a complex process of new blood vessel formation from previously existing vessels. This process plays a fundamental role in reproduction, development, and wound repair. In adults, endothelial cell proliferation is a highly regulated process established by a balance between angiogenic and angiostatic factors that are activated when necessary and then inhibited completely when the need is eliminated [35]. Cases of increased rate of endothelial cell proliferation are often linked with cancer and tumor development [36] which are known to be dependent on angiogenesis for growth and metastasis [37]. Vasculogenesis, on the other hand, refers to a process of* de novo* formation of blood vessels arising from migration, proliferation, and incorporation of angioblasts or endothelial progenitor cells (EPCs) from the bone marrow, usually occurring during embryogenesis [36]. The survival of endometriotic implants on the peritoneal membrane within the peritoneal cavity relies upon the establishment of blood supply for the provision of oxygen and nutrients to the developing lesions. Endometriotic lesions are densely vascularized, fueling the notion that angiogenesis and/or vasculogenesis may be involved [38]. Analogous to the process of vascularization of tumors, endometriosis may utilize mechanisms of both angiogenesis and vasculogenesis to establish its own vascular network to sustain its survival (Figure 1). Here, we discuss potential mechanisms exploited by the developing endometriotic lesions towards establishment of its own vasculature supply.

The endometrial fragments sloughed off from the endometrium of the uterus may harbour innate angiogenic potential due to the following characteristics. The human endometrium, composed of functionalis and basalis layer, is a unique organ that undergoes proliferation, differentiation, and regeneration with each menstrual cycle under the regulation of ovarian steroid hormones, estrogens, and progesterone. Along with the growth of the endometrium, the vasculature of the endometrium will experience proliferation and regeneration each cycle under the influence of the ovarian steroids, specifically E2. Shifren et al. [39] measured increased expression of vascular endothelial growth factor (VEGF) mRNA in the functionalis layer of the endometrium through proliferative and secretory phase of the menstrual cycle, indicating angiogenesis is in play. In the same study, E2 was responsible for the stimulation of VEGF expression from isolated human endometrial cells, as administration of E2 led to an increase in VEGF mRNA expression compared to the endometrial cells without E2 stimulus. Endometriosis is theorized to arise from implantation of endometrial fragments in the peritoneal cavity. With healthy endometrium showing innate angiogenic potential under the regulation of E2, it is evident that aberrantly regulated VEGF expression and E2 level may facilitate the neovascularization of endometriotic lesions that fuels its establishment in aberrant locations.

Indeed, VEGF plays a crucial role in facilitating the process of angiogenesis in endometriosis. It is a vasoactive substance involved in a variety of normal physiological processes including wound healing and revascularization of endometrium by mediating endothelial cell proliferation and migration. In tumorigenesis, VEGF concentration is typically correlated with increased vascularity in various types of tissue associated tumors (reviewed in [40]). In normal endometrium, VEGF mRNA and protein expression can be driven by hypoxia [41]. Not surprisingly, the PF of women with advanced stages of endometriosis contains higher concentrations of VEGF compared to women with mild endometriosis or healthy patients [42]. In addition, this elevated level of VEGF concentration in both PF and serum in endometriosis patients is positively associated with increased proliferative activity and microvessel density of the endometriotic lesions [43], indicating its involvement in the development of blood vessels. Various sources of VEGF have been indicated, including endometriotic lesions [44] and PF macrophages in endometriosis, which increase VEGF expression when treated with ovarian steroids such as E2 and progesterone [45], solidifying the notion that VEGF is involved in angiogenesis associated with endometriotic lesions. Other angiogenic cytokines including IL-1*β*, IL-6, and IL-8 will be further discussed in other sections of this review.

Vasculogenesis was generally accepted to be only prevalent during embryogenesis and that postnatal neovascularization of tissues occurred solely through angiogenesis [46]. The paradigm has shifted with the discovery of CD34+ and Flk1+ circulating endothelial progenitor cells (EPCs) in adult peripheral blood with phenotypic characteristics of endothelial cells* in vitro* [47]. This study in addition to the results published two years later definitively showed the presence and active involvement of bone marrow-derived EPCs in neovascularization of tissues including the endometrium [48]. Becker et al. (2011) confirmed the incorporation of the bone marrow-derived EPCs into the vasculature of the endometriotic lesion by transplanting GFP+ bone marrow-derived cells into mice with surgically induced endometriosis [49]. Laschke et al. (2011) further visualized the recruitment of the bone marrow-derived EPCs into the site of the endometriotic lesion development by elucidating the involvement of stromal cell-derived factor-1 (SDF-1) in the mobilization of bone marrow-derived EPCs into the lesions [50]. To confirm the chemotactic ability of SDF-1, Laschke et al. (2011) showed that, by antagonizing SDF-1 receptor—CXCR-4—with AMD3100, the number of recruited EPCs and the subsequent vascularization of endometriotic lesions significantly decreased. These results were confirmed by another study that demonstrated SDF-1 to be a chemokine capable of trafficking hematopoietic stem cells and EPCs whereby its focal concentration leads to increased vascularity of that region [51]. Our group recently demonstrated that blocking of SDF-1 in an alymphoid mouse model of endometriosis resulted in a decrease in endometriotic lesion vascularization and growth [52]. Collectively, these studies confirm that vasculogenesis in addition to angiogenesis is taking place, as demonstrated by the capacity of the lesion to mobilize and incorporate EPCs from the bone marrow into the vasculature of the lesions.

Furthermore, different types of immune cells are involved in the process of angiogenesis by producing proinflammatory and angiogenic cytokines and by increasing their concentration within the PF that bathes the endometriotic lesions (reviewed in [53]). Lin et al. [54] elucidated the importance of immune cells by demonstrating that angiogenesis of endometriotic lesions occurs after infiltration of VEGF secreting neutrophils and macrophages into the lesions as well as within the peritoneal cavity, indicating the essential role played by infiltrating leukocytes in the mouse model of endometriosis. In addition, dendritic cells (DCs) have shown their involvement in angiogenesis. A study conducted by Fainaru et al. [55] supports this argument by demonstrating increased perivascular distribution of VEGFR-2 expressing immature DCs in the endometriotic lesions with the ability to induce the migration of endothelial cells* in vitro.* The presence of DCs in the peritoneal cavity resulted in endometriotic lesion growth and vascularization of endometriotic lesion in this mouse model of endometriosis. In another study utilizing transgenic mouse model with conditional DC depletion (diphtheria toxin-treated B6.FVB-Itgax-hDTR-EGFP^tg^), researchers found that endometriosis lesions in DC depleted mice were significantly greater in size compared to control and showed decreased expression of CD69, a marker for T and natural killer cell activation. Based on these findings, it is apparent that DCs directly participate and regulate angiogenic process as well as subset of immune activation during endometriosis lesion development [55, 56].

Human endometrium has the unique ability to undergo cyclical proliferation and regeneration of the functionalis layer after physiological shedding of the endometrium. Thus, endometrial fragments exuded from the uterus will retain angiogenic capabilities in the peritoneal cavity. Postnatal neovascularization was once thought to be only possible in limited circumstances. It is now apparent that, in endometriosis vascularization, both angiogenesis and vasculogenesis are taking place at the site of the lesion. Under the regulation of E2, which augments expression of VEGF from the peritoneal macrophages, neovascularization of endometriotic lesion seems to mainly occur from the preexisting blood vessels of the peritoneal membrane under the process of angiogenesis. The complete elucidation of mechanisms underlying the process of angiogenesis remains complex due to other immune cells and mediators that are involved in neovascularization. In comparison, the process of vasculogenesis seems more concise, as demonstrated by studies that clearly showed the incorporation and recruitment of bone marrow-derived EPCs to the vasculature of endometriotic lesion. Indeed, neovascularization of the lesion utilizes both processes of angiogenesis and vasculogenesis. Knowing the mechanisms behind the establishment of vasculature will further aid in the development of therapies targeted towards lesion ablation, which may prove to be more beneficial compared to currently existing hormonal therapies used in treatment of endometriosis.

## 5. Immune Dysfunction and Endometriosis

Although endometriosis is common among women of reproductive age, the incidence of endometriosis is small compared to the occurrence of the retrograde menstruation that is experienced by most women of the same category. One hypothesis that arises then is that the women that develop endometriosis compared to those that do not have a defective immune system that is unable to recognize and properly mount immune response to the endometrial fragments within the pelvic cavity (Figure 1). It is speculated that endometrial fragments themselves acquire the ability to evade the immune system as they enter the pelvic cavity. We cannot exclude the possibility that both the fragments and the immune system are aberrant in women with endometriosis. In this section, we summarize the potential implication of the innate (macrophages, neutrophils, DCs, and NK cells) and adaptive immune cells (T and B cells) in the pathogenesis of endometriosis.

The menstrual endometrial fragments induce inflammation within the peritoneal cavity [57]. In response to the presence of these fragments, the sentinels of the immune system such as neutrophils and macrophages are among the first to be recruited to the area. Indeed, macrophage concentration and proportion are increased in the PF of women with endometriosis, and they are the primary contributors to the elevated proinflammatory and chemotactic cytokines found in the PF [58]. In addition to partaking in the growth of peritoneal implants, macrophages are a major source of angiogenic mediators including TNF-*α* and IL-8 [59]. Furthermore, macrophages are involved in the regulation of hypoxia-induced angiogenesis by producing VEGF [45]. Macrophage depleted Balb/C mice display endometriotic lesions that not only are smaller in weight and size compared to the control mice but also display reduced vascularization of the lesion [60], indicating that macrophages are involved in the process of growth and development of blood vessels. The same study, however, found that macrophage depletion does not prevent endometrial cells from implanting onto the peritoneal membrane, which suggests different mechanisms are involved in the process of implantation in the pathogenesis of endometriosis.

More recently, neutrophils have gained much attention and have been hypothesized to play an important role in the pathogenesis of endometriosis. Amongst most leukocytes implicated in inflammation, neutrophils have the shortest life span and contribute significantly to the resolution of inflammatory reaction. Neutrophils from disease-free women, when incubated with plasma or PF from women with endometriosis, displayed decreased rate of apoptosis compared to control women [61]. This study clearly indicated a potential existence of antiapoptotic factors in the plasma and PF in women with endometriosis that is not as concentrated in women without the disease. IL-8 was one of the potential factors investigated given its well established role as a proinflammatory cytokine and a key factor involved in the chemotaxis of neutrophils during inflammation. However, treatment with anti-IL-8 antibody prior to adding PF or plasma from endometriosis patients did not have marked difference in apoptosis rate of neutrophils, which may indicate the presence of other factors that may be in play. This study also showed that neutrophils from women with endometriosis may be more resistant to spontaneous apoptosis than the neutrophils from control. These findings further contribute to the notion of dysregulated immune response in women with endometriosis.

Dendritic cells (DCs), a type of antigen presenting cells (APCs), are paramount in the activation of adaptive immunity through antigen presentation to naïve T cells. Dendritic cells, like macrophages, differentiate from monocytes in the presence of IL-4/GM-CSF* in vitro*. However,* in vivo,* DCs only require as low as picomolar to nanomolar concentrations of antigens for presentation; thus they are powerful in detecting and initiating adaptive immunity on foreign or self-antigen [62]. Once an antigen is captured, maturation of DCs occurs, whereby they gain the ability to activate the naïve T cells into cytotoxic or T helper state. DCs also play a vital role in the prevention of autoimmunity by acting as mobile sentinels that bring self-antigens to the lymphoid organ-resident naïve T cells to promote induction of self-immunity [62]. Immature DCs are nonexistent in the peritoneal membrane of healthy women; however, they are found within the endometriotic lesions and the surrounding peritoneal membrane of women with endometriosis [63]. Furthermore, the numbers of mature DCs are significantly decreased in both functionalis and basalis layers of endometrium of women with endometriosis throughout the menstrual phase compared to the healthy endometrium [63]. The implication of low distribution of immature DCs in the endometrium or the diminished numbers of the mature DCs in both functionalis and basalis layer throughout the menstrual phase in women with endometriosis is unclear; however they likely promote angiogenesis of the lesion. Furthermore, conflicting findings from two independent investigations obscure the role of DCs in the pathogenesis of endometriosis. Stanic and colleagues (2014) reported on the depletion of DCs leading to the growth of the endometriotic lesion [56], whereas Pencovich and colleagues (2014) reported on the exact opposite—the depletion of DCs attenuated the development of endometriosis [64]. One possible explanation of the differing results despite utilizing a similar transgenic mouse model using diphtheria toxin (DT) (B6 FVB-Itgax-hDTR-EGFP^tg^) [56] and B6.FVB-Tg(Itgax-DTR/EGFP) [64] may be that the time for lesion retrieval was delayed by 3 days and that the receptor for DT was human [56] compared to simian DT receptor [64]. Investigations into the role of DCs need further fine-tuning as they appear to play a crucial role in the pathogenesis of endometriosis, in particular by promoting angiogenesis and inducing activation of adaptive immunity.

Diminished cytotoxicity of natural killer (NK) cells within the peritoneal cavity has also been well documented. Somigliana et al. [21] reported on the presence of immunosuppressants in both the conditioned media (CM) of normal endometrial stromal cell and of endometriotic stromal cells. This implies that the normal endometrium harbours innate immunosuppressive ability against cytotoxic activity of NK cells, possibly to allow the implantation of the embryo. In women with endometriosis, this immunosuppressive effect on NK cell cytotoxicity was greater, which in peritoneal environment may allow endometrial fragments to develop into lesions [21]. Such reduction in NK cytotoxicity seems to stem not due to decrease in quantity but due to functional defect, as the number of NK cells did not seem to differ between patients and control [65]. Recently, IL-6 in PF of women with endometriosis has been identified as a possible immunosuppressant towards NK cell cytotoxicity against autologous endometrial fragments [66]. These studies indicate possible association of NK cells with immune dysfunction in endometriosis.

The role of adaptive immunity, particularly T helper cells and B cells, is less defined. In brief, cell mediated immunity is facilitated by T helper type 1 cells (Th1) that target intracellular pathogens whereas humoral-mediated or T helper type 2 cells (Th2) target extracellular pathogens and are involved in B cell activation and antibody secretion. In women with endometriosis, a polarization towards Th2 cells has been observed due to strong intracellular expression of IL-4 and absence of IL-2 from the lymphocytes isolated from the ectopic lesions [67]. Furthermore, increased activation of B cells was also detected from the eutopic endometrium as well as the lesions compared to healthy endometrium. Indeed, endometriosis is sometimes categorized as an autoimmune disease due to anti-endometrial antibodies being detected in the serum of women with endometriosis [68]. The balance of T helper cells in women with endometriosis remains controversial with some studies reporting diminished activation of both Th1 and Th2 in the PF of women with endometriosis [69]. Furthermore, in contrast to Szyllo et al., another study failed to detect any difference in the intracellular concentration of IFN-*γ* and IL-4 from PF lymphocytes between endometriosis patients and healthy controls [70]. In particular, genome-wide gene array and immunostaining for B (CD20+) and T (CD3+) cells in ovarian endometriomas failed to detect gene expression and presence of either of cell types despite overexpression of B lymphocyte stimulator [71]. Contradictions in results between independent studies are likely due to different experimental methods and thus warrant further investigation.

## 6. Cytokines and Chemokines in Endometriosis

Cytokines are the main mediators and communicators of the immune system. Although these polypeptides are mostly produced by immune cells, most nucleated cells also produce cytokines, albeit in lesser quantities. Immune cells use cytokines to coordinate the host response to infection or trauma via autocrine and paracrine signaling. Based on their immune-regulatory role, cytokines are broadly classified as either pro- or anti-inflammatory. Proinflammatory cytokines such as interleukin-1 (IL-1), tumor necrosis factor alpha (TNF-*α*), interferon gamma (IFN-*γ*), and granulocyte-macrophage colony-stimulating factor (GM-CSF) primarily initiate and amplify the inflammatory response to infection or trauma by signaling the recruit of additional immune cells and proinflammatory mediators to the site of injury. Anti-inflammatory cytokines such as IL-4, IL-6, and IL-10 primarily regulate the intensity and duration of the inflammatory response by suppressing the effects of proinflammatory cytokines, although some have inflammatory roles as well [72]. Chemokines, such as monocyte chemoattractant protein-1 (MCP-1), IL-8, and stromal cell-derived factor-1 (SDF-1), are capable of recruiting immune cells to the site of injury and stimulate them to produce additional cytokines. The cascade of events that comprises the inflammatory response is an important aspect of endometriosis development. The normal immune response to pathogens or injury entails a delicate balance of inflammatory and anti-inflammatory cytokines and regulators in order to be effective and remain safe for the host. Thus, cytokine dysregulation is recognized as an important aspect of the pathogenesis of numerous conditions, including endometriosis. Previous studies have found increased total leukocyte concentrations in addition to noticeable disruption of the immune activity in women with endometriosis [58]. Peritoneal fluid contains higher concentration of proinflammatory and angiogenic cytokines presumably produced from immune cells such as macrophages and from the lesion itself, which contribute to the pathogenesis of endometriosis (Figure 1). Furthermore, the PF from women with endometriosis has components that polarize monocytes into macrophages instead of DCs, which are potent antigen presenting cells compared to macrophages even in the presence of dendritic cell polarizing cytokines* in vitro* [73]. In this section, we examine various cytokines and chemokines that seem to play a significant role in the establishment and survival of lesions in endometriosis.

IL-1 is an acute phase inflammatory cytokine that exists in three main forms—IL-1*α*, IL-1*β*, and IL-1 receptor antagonist (IL-1Ra) [72]. The release of IL-1*α* and IL-1*β* by mononuclear and epithelial cells in response to injury leads to inflammation, while IL-1Ra release attenuates this response by blocking IL-1*α* and IL-1*β* binding. Various studies have reported higher concentrations of IL-1*α* [74], IL-1*β* [75], and total IL-1 [76] in the PF of women with endometriosis compared to normal women, thus supporting the notion of a local inflammatory environment in endometriosis. This idea is further supported by studies reporting impaired expression of the soluble decoy receptor IL-1-RII in the endometrium and PF of women with endometriosis [74, 77], which would help attenuate the effects of IL-1*α* and IL-1*β*. Similarly, decreased levels of IL-1Ra have been reported in the PF of patients with early stage endometriosis [74]. These results may reflect an initial but failed attempt to attenuate the local inflammation caused by endometrial fragments in the pelvic cavity. The fact that shed endometrial fragments would trigger such a strong inflammatory response points to either a reduced capacity of immune cells to clear these fragments or a potential autoimmune condition that would cause peritoneal resident immune cells to be more sensitive to endogenous damage signals. A study by Bergqvist and colleagues found that endometriotic lesion expresses higher levels of IL-1*β* than eutopic endometrium of both normal women and women with endometriosis, which indicates that the inflammation in endometriosis is locally induced.

Tumor necrosis factor alpha (TNF-*α*) is the most studied protein of the TNF family and is primarily produced by activated macrophages, NK cells and Th1 cells [72]. TNF-*α* appears to act synergistically with IL-1, as they both activate the canonical NF-*κ*B inflammatory pathway. Harada and colleagues found increased levels of TNF-*α* in the PF of women with endometriosis and detected a positive correlation between TNF-*α* concentrations and endometriotic lesion size [78]. Others have also reported higher levels of TNF-*α* in the endometrium and PF of women with endometriosis [76, 79] but only in mild or early stages of the disease, which suggests that TNF-*α* plays a role in the early stages of endometriosis when the lesions are establishing. Interestingly, both TNF-*α* and IL-1 are capable of inducing the expression of cyclooxygenase-2 (COX-2), the enzyme that regulates the synthesis of prostaglandin E_2_ (PGE_2_) [80]. Unlike the constitutive COX-1 enzyme, COX-2 is undetectable under normal conditions and only becomes upregulated in response to infection or injury. In women with endometriosis, COX-2 has been found to be overexpressed in isolated peritoneal macrophages, but not in isolated peripheral macrophages [81], which supports the idea that local inflammatory factors are responsible for the upregulation of COX-2 in macrophages. Furthermore, PGE_2_ itself can induce COX-2 expression, creating a positive feedback cycle that promotes inflammation and pain via overproduction of PGE_2_. PGE_2_ can also attenuate macrophage cytotoxicity and promote local estrogen synthesis, cell proliferation, and angiogenesis (reviewed in [80]).

IL-6 possesses prominent inflammatory and anti-inflammatory functions, which makes it challenging to understand its full role in endometriosis. Although mainly produced by macrophages, Th1 cells and B cells, IL-6 can be produced by fibroblast and endothelial cells as well [72]. In endometriosis patients, the PF levels of IL-6 have been found to be increased compared to normal women [78, 82] and positively correlated with the size and number of endometriotic lesions [78]. Bergqvist and colleagues reported higher levels of IL-6 in both endometriotic lesion and eutopic endometrium from endometriosis patients compared to normal women [83]. IL-6 also seems to increase in concentration in more advanced stages of endometriosis [84, 85]. The high levels of IL-6 could be produced by the increased number of macrophages that infiltrate the peritoneal cavity in endometriosis. However, peritoneal mesothelial cells have also been shown to synthesize IL-6 in response to IL-1*β* and TNF-*α* [86]. These last two are mainly produced by macrophages, which are presumably recruited to the peritoneal cavity to help clear the endometrial fragments. Increasing levels of IL-1*β* and TNF-*α* would induce the production of IL-6 by peritoneal mesothelial cells, which would further contribute to the local inflammation observed in endometriosis.

IL-10 is a known anti-inflammatory cytokine capable of inhibiting the synthesis of inflammatory cytokines IFN-*γ*, IL-2, IL-3, TNF-*α*, and GM-CSF [72]. Ho and colleagues found increased levels of IL-10 in the PF of women with endometriosis compared to normal women [69]. A more recent study by Suen and colleagues showed that serum levels of IL-10 were higher in endometriosis patients compared to both healthy subjects and subjects with other gynecological diseases [87]. They also demonstrated that, in a C57BL/6 mouse model of surgically induced endometriosis, endometriotic lesion growth can be promoted or decreased by administering or depleting IL-10, respectively. The increased concentration of IL-10 has been implicated in the decreased cytotoxicity of NK cells observed in endometriosis [21] and supports the notion that local cytokine dysregulation allows endometrial fragments to implant in the peritoneal cavity.

IL-8, also known as CXCL8, is a potent neutrophil chemotactic factor with proinflammatory and angiogenic effects [88]. Studies have found higher levels of IL-8 in the PF of women with endometriosis [82, 89], but not in the serum [82] or peripheral blood [90]. These results point to a local dysregulation of IL-8 in endometriosis. Others have reported a significant correlation between IL-8 levels and disease stage [90, 91], with higher levels of IL-8 reported in early stages of endometriosis compared to more advanced stages [90, 91]. Akoum and colleages reported that IL-1 can induce IL-8 secretion in isolated epithelial and stromal endometriotic cells and that E2 stimulation enhances endometriotic cell responsiveness to IL-1 [92]. Given that endometriosis is an estrogen-dependent condition, IL-1 mediated induction of IL-8 could link local estrogen overproduction with the recruitment of neutrophils to the site of lesion implantation. Interestingly, mesothelial cells isolated from the PF of endometriosis patients have been reported to produce IL-8 in response to IL-1*α* and TNF-*α* stimulation [89]. Furthermore, in a study by Li et al., two human endothelial cell lines were stimulated with recombinant human IL-8, which resulted in endothelial cell proliferation and capillary tube organization, inhibited apoptosis, enhanced antiapoptotic gene expression, and upregulated MMP-2 and MMP-9 expression [93]. This evidence points to a crucial involvement of IL-8 in the establishment and maintenance of endometriotic lesions, likely via the activation of angiogenic factors normally released in response to injury.

Monocyte chemoattractant protein-1 (MCP-1) is a proinflammatory chemokine implicated in the activation macrophages, monocytes, and lymphocytes [88]. MCP-1 has been detected in high concentrations in the PF of women with endometriosis [82, 94] and has been reported to increase with disease severity [94]. Although mostly produced by peritoneal macrophages [95], MCP-1 production has been detected in the glandular epithelium and stromal macrophages of endometriotic lesions [96]. Arici and colleagues reported that mesothelial cells isolated from the PF of women with endometriosis not only produce MCP-1 in response to IL-1*α* and TNF-*α* stimulation, but also constitutively produce the cytokine as well. They also found that, in healthy women, MCP-1 production was correlated with stage of menstrual cycle, where the PF of healthy women had higher MCP-1 levels during the proliferative phase compared to the secretory phase. These results point towards a responsiveness of MCP-1 to ovarian hormones. In a later study, the same group demonstrated that MCP-1 production and expression in isolated endometrial stromal cells are inhibited by E2 in a dose dependent manner [97]. Adding progesterone caused a slight decrease that did not differ significantly from E2 treatment alone. They also determined that endometriotic lesion can be stimulated to produce MCP-1 by IL-1*β* and that this response is enhanced by E2_._ These results not only show the significant involvement of MCP-1 in the development of endometriosis, but also reveal the complex interplay between the endocrine and immune systems by showing the crucial role of estrogens in enhancing the chemokine-induced recruitment of immune mediators to the endometriotic lesion sites.

## 7. Conclusion

Current evidence indicates that immunological factors are significantly involved in the pathogenesis of endometriosis (summarized in Figure 1); however it is still unclear if the dysfunctional immune response seen in women with endometriosis is the cause for endometriosis development. The aberrant immune cell behavior seen in women with endometriosis helps the implantation and survival of endometriotic lesions via upregulation of inflammatory pathways that are normally deployed in response to infection or trauma. Endometrial cells from women with endometriosis, which are precursors to endometriotic lesions, are able to exploit the promotion of vasculogenesis and angiogenesis mediated by the inflammatory response they trigger. In this process, both immune cells and the local peritoneal tissue orchestrate such processes using cytokine signaling. Although the role of cytokines and chemokines in endometriotic lesion survival is well established, it remains poorly understood. Part of this is due to the fact that these modulators are highly pleiotropic proteins that also exhibit considerable redundancy in their functions. Because of this, it is difficult to conclusively determine how they influence the pathogenesis of endometriosis. The main question continues to be whether cytokine dysregulation is one of the triggers in the development of endometriosis or if it arises after endometriosis has developed through other mechanisms. These aberrant immune responses are further exacerbated by the unique hormonal environment in which they develop. However, it is evident that additional mechanisms are involved in triggering these aberrant immune responses. Based on the evidence pointing to the aberrant modulation of immune factors contributing to endometriotic lesion implantation and survival, there are debating views on whether to classify endometriosis as an inflammatory condition or an autoimmune disorder. More research is needed not only to reach a better understanding of this condition, but also to improve our current approaches in its treatment.
